# The Use of Genomics in Conservation Management of the Endangered Visayan Warty Pig (*Sus cebifrons*)

**DOI:** 10.1155/2016/5613862

**Published:** 2016-03-16

**Authors:** Rascha J. M. Nuijten, Mirte Bosse, Richard P. M. A. Crooijmans, Ole Madsen, Willem Schaftenaar, Oliver A. Ryder, Martien A. M. Groenen, Hendrik-Jan Megens

**Affiliations:** ^1^Animal Breeding and Genomics Centre (ABGC), Wageningen University, 6708 PB Wageningen, Netherlands; ^2^Department of Animal Ecology, Netherlands Institute of Ecology (NIOO-KNAW), 6708 PB Wageningen, Netherlands; ^3^Veterinary Services, Rotterdam Zoo, 3041 JG Rotterdam, Netherlands; ^4^San Diego Zoo Institute for Conservation Research, Escondido, CA 92027, USA

## Abstract

The list of threatened and endangered species is growing rapidly, due to various anthropogenic causes. Many endangered species are present in captivity and actively managed in breeding programs in which often little is known about the founder individuals. Recent developments in genetic research techniques have made it possible to sequence and study whole genomes. In this study we used the critically endangered Visayan warty pig (*Sus cebifrons*) as a case study to test the use of genomic information as a tool in conservation management. Two captive populations of* S. cebifrons* exist, which originated from two different Philippine islands. We found some evidence for a recent split between the two island populations; however all individuals that were sequenced show a similar demographic history. Evidence for both past and recent inbreeding indicated that the founders were at least to some extent related. Together with this, the low level of nucleotide diversity compared to other* Sus* species potentially poses a threat to the viability of the captive populations. In conclusion, genomic techniques answered some important questions about this critically endangered mammal and can be a valuable toolset to inform future conservation management in other species as well.

## 1. Introduction

The list of threatened and endangered species is growing rapidly, due to various anthropogenic causes. Current management of endangered species includes* in situ* and* ex situ* measurements.* In situ*, that is, within the range of the species, most conservation actions focus on habitat protection (protected areas), law enforcement (for reducing threats), and sometimes translocations or reintroductions [1]. For* ex situ* management, that is, outside the range of the species, such as in zoos or conservation centres, actions are mostly focused on keeping the population viable (both demographically and genetically) and as similar to the wild ancestor populations as possible (i.e., prevent adaptation to captivity [2]).

Despite the successes achieved with these approaches, there are also several challenges, for example, in prioritizing species for conservation. In trying to solve this problem, the International Union for the Conservation of Nature (IUCN) in 1994 initiated a scientific approach to categorise endangerment of species: the Red List of Threatened Species. This comprehensive list is currently a leading reference for governments, NGOs, and research institutions to decide on how to spend valuable resources for species conservation [1]. However, assessing a species properly is time-consuming and requires much information, which is often lacking. Additionally, not all species can be easily observed, creating a bias in the Red List towards the “easier observed” species [3]. If a species is not assessed well (or not assessed at all) the conservation actions planned for it might also miss their purpose. Another difficulty is the time that is needed to assess a species, understand its situation, implement suitable conservation actions, and wait for them to have an effect. With the current rate of extinction, this time might not always be available.

Nowadays, both as a legacy from earlier times and as a recent conservation measure, many threatened and endangered species are present in captivity [5]. These populations are often managed within a breeding program with the highest priority. This* ex-situ* management is mostly based on a pedigree, which requires information on relatedness between individuals. Especially for the individuals in the founder generation, however, information on relatedness is often missing. This causes breeding programs to make the potentially dangerous assumption that all founders are unrelated individuals [6]. If this assumption is not met, it can lead to unintended inbreeding events causing loss of genetic diversity and deleterious effects [7].

The development of genomic techniques has opened up many new opportunities for both breeding and population management and has caused a revolution in the field of commercial animal breeding [8, 9]. The recent developments make it possible to sequence and study the whole genome of individuals (genomics or next generation sequencing, NGS, [10]).

The main improvement of applying NGS, with respect to genetic methods such as microsatellites and SNP-arrays, is the enormous increase in loci that can be studied [11]. This allows for a more detailed study of previous research questions and opens up a completely new range of applications. For example, the possibility to study functional genetic variation (as opposed to neutral variation) interactions within the genome and interactions between the genome and the environment [12–15]. Although whole genome sequencing is still costly relative to targeted genotyping technologies, it rapidly becomes less expensive. Therefore it is expected that in the near future NGS techniques will be feasible for noncommercial research areas such as conservation biology as well [12, 16, 17].

The aim of this study is to apply currently available genomic techniques on whole genome sequencing data of a critically endangered mammal to test their application in species conservation and (captive) population management. It is expected that the outcomes of this study can be of direct use for conservation management of the species both* in situ* and* ex situ*.

**Figure 1:**
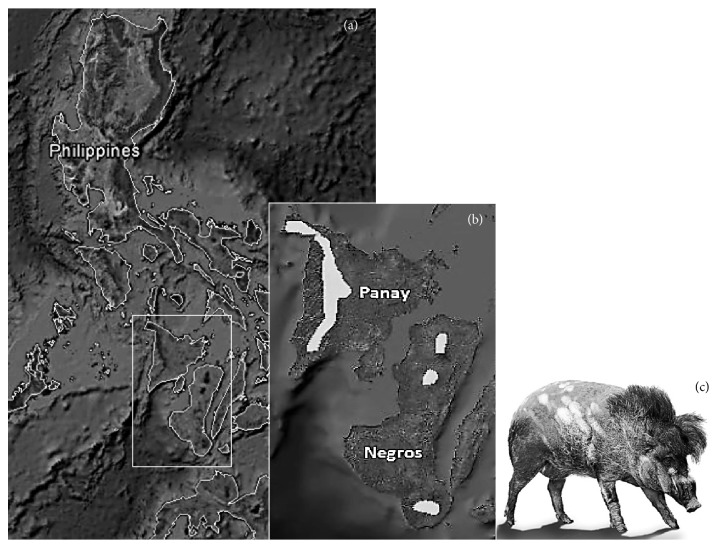
(a) Location of the islands Negros and Panay within the Philippine archipelago. At its smallest point the Guimaras Strait between them is 10 km wide. (b) Negros and Panay with the range of* Sus cebifrons* indicated in light-grey [4]. (c)* Sus cebifrons *individual (DVDW Photography).

As a case study, the critically endangered Visayan warty pig (*Sus cebifrons*) was used.* S. cebifrons* is endemic to the Philippines and mainly lives on the islands of Negros and Panay (10°00′N, 123°00′E and 11°09′N, 122°29′E, resp.) (Figure 1). On Negros it is estimated by experts in the field that only 200–500 individuals remain, while this estimate is 500–>1000 for Panay. The main threats to the populations are habitat loss due to commercial logging operations and slash-and-burn farming, hunting for meat, negative reputation (crop damage), and genetic contamination via hybridization with free-ranging domestic and feral pigs (*Sus scrofa*) [4]. Despite several* in situ* conservation measures [4, 18] the population remained small with a decreasing trend. Therefore it was decided to create two captive populations in zoos (*ex situ*). San Diego zoo received a breeding group from a conservation centre on Panay and in 2004 another group of 8 founders (4 male, 4 female) was moved from conservation centres on Negros to Rotterdam zoo in the Netherlands to found a European captive population [4]. The Visayan warty pig fits our aim perfectly because of its level of endangerment, the availability of genomic data, and the uncertainty about the degree of divergence between the two island populations. In addition, because the captive populations were founded recently, it may be possible to draw conclusions on the situation of the wild population from the genomic information of captive individuals. The main questions assessed in this study concern the presence of a substructure between the two island populations, the genetic status of the species including the (historic) demography that explains this status, and the assumption of unrelatedness for the founder individuals of the captive population.

## 2. Methods

### 2.1. Generation of Data

Demographic information on the captive populations was gathered from studbook files kept by regional (i.e., EU or US) studbook coordinators and analysed in the software program PMx [19]. From these files the relationship between the sampled individuals was extracted.

DNA samples were previously sequenced [20, 21]. The data is deposited at the European Nucleotide Archive (ENA) under accession numbers PRJEB9326 and ERP001813 for* S. cebifrons* and other individuals, respectively (http://www.ebi.ac.uk/ena/). Seven complete whole genome sequences of* Sus cebifrons individuals *were available, of which two came from Rotterdam zoo and five from San Diego zoo (Table 1).

Resequencing data of* Sus cebifrons *individuals was aligned against the* Sus scrofa* reference genome (version 10.2 [22]) using the unique alignment option of MOSAIK aligner [23] and variants (Single Nucleotide Polymorphisms or SNPs) were called using SAMtools mpileup (version 0.1.19 [24]). Only variants with a read-depth between 0.5 and 2.0 times the average (i.e., between 5x and 20x) were selected and stored in variant call format (.vcf) using VCFtools (version 0.1.1 [25]). Unless otherwise stated, these “filtered variants” were used for all analyses.

### 2.2. Population Structure

To assess the phylogenetic relationship between individuals of* S. cebifrons* and between* S. cebifrons* and other species/populations within the genus* Sus*, a phylogenetic analysis was carried out. Variants were called for individuals using ANGSD (minimal mapping quality 30, minimal base quality 15, and SNP *p* value of 1*e* ^−6^ [26]). Pairwise distances between individuals were calculated using PLINK 1.9 [27, 28], and hierarchical clustering was done by neighbour joining [29].

To obtain insight in whether or not the individuals from the two different islands were admixed, we used the software Admixture [30]. Only biallelic variant sites from the filtered variants were used as input file. We initially set *K* (i.e., the number of source populations) to 2 because the individuals came from two different islands, but tested for different *K*-values (1, 2, 3, 4, 5, 6, and 7) by calculating cross-validation errors.

### 2.3. Demographic History

To derive an estimate of the historic effective population size of the population and possibly gather evidence for a population substructure, a Pairwise Sequential Markovian Coalescence (PSMC) model was used [31]. This software uses the time to most recent common ancestor of a diploid genome (determined by looking at the density of heterozygotes) to estimate the effective population size (Ne) in the (distant) past. The individual whole genome consensus sequence, called by SAMtools, was used as an input for this analysis. We used a generation time of five years (in concordance with the studbook files that showed a generation time for the captive population of 4,5 years) and a default mutation rate of 2,5 × 10^−8^ [22].

Demographic history of the individuals was studied by analysing regions of homozygosity (ROHs). ROHs can be informative for the level of inbreeding of a population; long ROHs are indicative for recent consanguineous matings while short(er) ROHs indicate more distant inbreeding as ROHs will break down over time due to recombination and mutation [32]. ROH abundance and length over time therefore depend on recombination and mutation rate [20]. To identify the ROHs present in an individual we used a sliding window approach with bins of 10.000 bp [20]. We filtered the variants for read-depth (0.5–2.0 times the average) to exclude sites with a low read-depth (low reliability) and sites with a very high read-depth (possible sequencing errors or copy number variants). A correction for missing sites was done, by scaling the number of identified SNPs up from number of covered sites to the total bin length. Bins with less than 1000 sites covered (<10% of total bin) were excluded from analyses. Sex chromosomes were also excluded from analyses as it is known that the recombination landscape of these chromosomes is different from the autosomes [20]. Adapted from Bosse et al. [20] we defined a ROH as a region of at least twenty consecutive bins with a number of SNPs per bin <0.33 of the genomic average. The average number of SNPs per bin (nucleotide diversity) outside ROHs was used as a measure of genetic diversity present in an individual. All these measures were also done for other* Sus* species for comparison (description of other species is in Supplementary Material, Table S1, available online at http://dx.doi.org/10.1155/2016/5613862).

Besides nucleotide diversity outside ROHs, all filtered variants were analysed with the Ensemble Variant Effect Predictor tool (VEP [33]). This tool was set to look for variants in coding regions only. Nonsynonymous variants were also annotated with SIFT (Sort Intolerant From Tolerant [34]) scores. SIFT scores range from 1 (“tolerated”) to 0 (“deleterious”). A site is classified as deleterious when the variant in the genome leads to a different amino-acid in a protein, which in turn leads to the protein having different characteristics, for example, in shape and function. A SIFT score close to zero infers that the identified SNP is likely to have an effect, but the nature and extent of the effect cannot be ascertained. Therefore, the term “not-tolerated” (as opposed to “tolerated”) will be used throughout this study to refer to sites with a SIFT score of (or close to) zero. For the VEP analysis and subsequent partitioning of variants between individuals and islands, variants were called again based on the positions identified previously, based on individually called genotypes. A multi-individual VCF was thus constructed using SAMtools mpileup (version 0.1.19 [24]). Only SNPs that were not fixed differences between* Sus scrofa* and* S. cebifrons* were retained. Furthermore, the minor allele count for variants to be considered was 2, to remove spurious allele calls as much as possible. Subsequently, variants were annotated using VEP based on Ensembl v83.

## 3. Results

### 3.1. Population Structure

**Figure 2:**
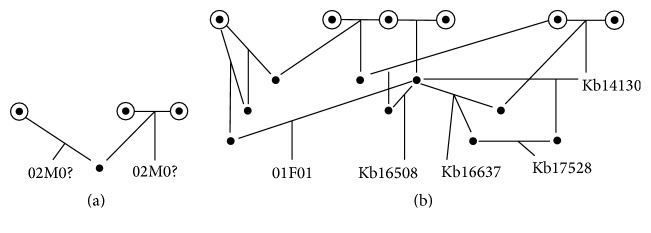
Part of the pedigree of the captive* Sus cebifrons *populations in Europe (a) and the US (b) reconstructed from the studbook files. Dots represent individuals, circles indicate founders (individuals with parents indicated as “WILD” in the studbook file), and sampled individuals are represented by their number as described in Table 1. For the two individuals from Rotterdam zoo, it is not known which sample is from which individual, and therefore they are indicated with a question mark.

**Figure 3:**
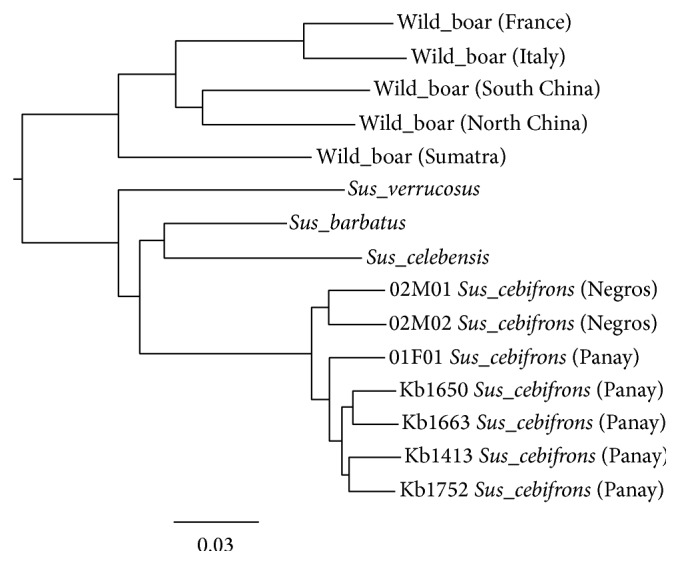
Neighbour joining, midpoint rooted phylogenetic tree of seven* Sus cebifrons* individuals, including other species for comparison (for description, see Table S1 in Supplementary Material). Wild boar samples represent different populations of* Sus scrofa*. Within the* Sus cebifrons* cluster, a split is visible, separating the two individuals from Negros (top) and the five individuals from Panay. The two smaller clusters within the Panay individuals are caused by relatedness between the sequenced individuals (Figure 2).

The pedigree of the captive populations for both breeding programs was extracted from the studbook files (partly visualized in Figure 2). From this pedigree, the inbreeding coefficient (*F*) was calculated for the sampled individuals (Table 2). The phylogenetic analysis showed two clusters within* Sus cebifrons *(Figure 3), separating the two island populations. The time of the split between these two clades of* Sus cebifrons* is comparable to the split between the two different European wild boar populations, which was estimated at about 1 million years ago [22]. Additionally, the Admixture analysis, when forced to use a *K*-value of 2, also identified the two island populations. However, the cross-validation error was lowest for a *K*-value of 6 (Supplementary Material, Table S2). These results both indicate that a substructure is present, at least to some extent.

### 3.2. Demographic History

**Figure 4:**
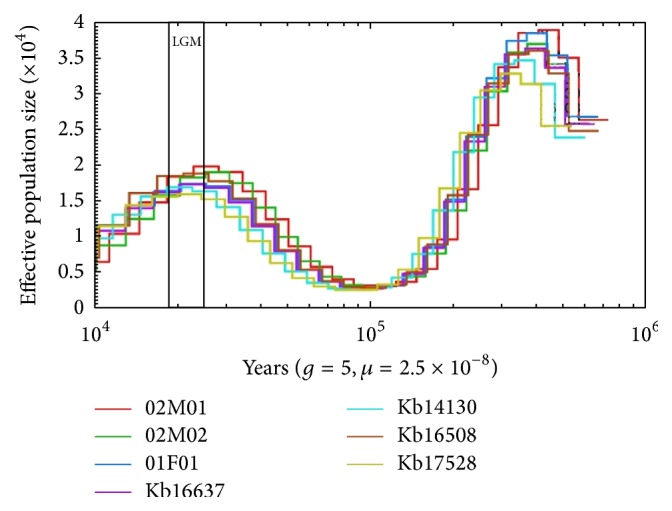
Estimated effective population size of* Sus cebifrons* based individual genomes, generation time (i.e., 5 years), and mutation rate (2.5*∗*10^−8^) from 10.000 (left) to 1.000.000 (right) years ago. The red and green lines indicate individuals from Rotterdam (originating from Negros island); the other individuals originated from San Diego (Panay island). A severe population bottleneck is found in all individuals around 100.000 years ago. The Last Glacial Maximum (LGM) occurred roughly 17.000 years ago (black rectangle).

The PSMC analyses did not show a divergence between the two islands; all individuals showed a similar pattern of historic effective population size. As the PSMC shows the historic effective population size between 10.000 and 1.000.000 years ago; this indicates that the substructure found in the phylogenetic and Admixture analyses arose only recently. The PSMC results also show two severe bottlenecks in all populations, inferred from the individual genomes, one occurring around 100.000 years ago, and another more recent one which coincides with the end of the Last Glacial Maximum (LGM; Figure 4). Both bottlenecks are also present in most other* Sus* species (Supplementary Material, Figure S1 [35]).

For the demographic analyses we also identified regions of homozygosity (ROHs) and nucleotide diversity. On average we found 117 ± 51 (average ± sd) ROHs in the* Sus cebifrons* individuals. This average was lower for Negros individuals than for Panay individuals, 55 ± 29 and 142 ± 31, respectively (Table 2). The average length of the identified ROHs was 1.9 ± 0.7 Mb. Here also a lower value for the Negros individuals was found, with 1.3 ± 0.7 Mb compared to 2.2 ± 0.6 for the Panay individuals. These numbers are similar as those found for other* Sus *species analysed using the same criteria (Table 2). In most* Sus cebifrons *individuals, the largest proportion of the genome was covered by ROHs in the longest category (Supplementary Material, Figure S2). However, most ROHs in all* Sus cebifrons* individuals fell within the shortest length category of 0.2–0.5 Mb. Although logic predicts the longest category would cover the largest proportion of the genome, a high number of short ROHs could easily cover a proportion of the genome larger than a few long ROHs. This is also shown by individuals of other* Sus *species (Supplementary Material, Figure S3). The large proportion of coverage by long ROHS is an indication of recent inbreeding [20].

Nucleotide diversity outside the ROHs did not differ between the islands and was on average 12 SNPs per bin (10 kb) in all* Sus cebifrons* individuals (Table 2) and seemed to follow a normal distribution (Supplementary Material, Figure S4). The other individuals showed on average 23 SNPs per bin (Table 2). The very low nucleotide diversity outside the ROHs is probably a direct effect of the extreme bottleneck found in the PSMC analysis, as a bottleneck generally causes rapid loss of genetic variation.

For variant effect prediction, 4679012 variants were retained. Of these, 38321 were exonic, 11532 were nonsynonymous but classified as tolerated according to SIFT predictions, and only 3884 were predicted to be not-tolerated (Table 3). Only a fraction—around 15%—of the variants was specific to either one of the islands based on the small population sample surveyed here (Supplementary Material, Table S3).

## 4. Discussion

The genomic analyses showed that, at least to some extent, a substructure is present between the two island populations of* Sus cebifrons*. This was visible in the results of the Admixture analysis and in the presence of island-specific variation. However, the PSMC analysis showed a similar demographic history for all individuals, regardless of their source population, suggesting one population of origin for the sampled individuals. This indicates that the present structure only arose recently in evolutionary terms (as the PSMC analysis provides estimates for 10.000–1.000.000 years ago). This hypothesis is supported by the relatively recent split in the phylogeny, suggesting that the split between European wild boar populations predated the split between the* Sus cebifrons* populations on both Philippine islands. For comparison, the split between European and Asian wild boar took place about 1 million years ago but the populations are considered one species [22]. Further evidence for a very recent split between the populations is the large proportion of shared variants between islands and thus the small fraction of island-specific variation.

The extremely low nucleotide diversity found in the analysed individuals, as compared to other species, is probably a result of the bottleneck visible in the PSMC result. The number of short ROHs present in the genomes is indicative of past inbreeding. The data also showed long ROHs that are signs of inbreeding in recent generations. Because both captive populations in the EU and US are under strict management to minimize inbreeding, it is plausible that some relatedness was already present in the founders of the two captive populations. The small population sizes present at the islands are indicative of this as well. In addition, it is not clear whether the founders were a representative sample of the island populations. It is clear from our results that the assumption of founder-unrelatedness was violated.

In the captive populations in the US and EU, numbers have increased rapidly since the founder generation. However, because reproduction was not equally successful for all individuals, the amount of genetic diversity present now is not the maximum that could have been retained. Furthermore, the extremely low nucleotide diversity and the signs of recent inbreeding (long ROHs) found in the current generation potentially threaten the viability of the captive populations. If populations are to be kept separately in the future, inbreeding in each of the populations has to be limited. However, with regard to the problems mentioned above, based on the similarity in demographic history of the individuals, it might be prudent to decide to merge the two breeding programs in order to increase the viability of the total captive population and the probability of reintroduction. By doing this the genetic diversity, and with that the potential for adaptation, will increase. Moreover, given the extremely shallow genetic divergence between the islands, problems of outbreeding depression are not expected.

A decision for merging captive populations cannot be based on the present study alone. Further research should focus on deleterious load present in both captive populations. Purging in these naturally small populations may have removed variants that are deleterious in homozygous state [36]. The ratio between heterozygous and homozygous states in the not-tolerated variants found in this study is an indication that purging has removed some variants from the populations (Supplementary Material, Table S4). Merging the two captive populations would increase the frequency of these variants, which could have deleterious effects. The decision to merge two captive populations should therefore be made with caution. Information on, for example, the heterozygous/homozygous ratio of variants can help in making informed decisions.

Although it is not the focus of this study, the same genomic methods as described here can be used to select individuals for breeding [21]. Individuals can be selected based on deleterious load or identity-by-descent (IBD) segments. It has been shown that inbreeding measures based on ROHs are more reliable than inbreeding estimates from a pedigree [32]. Also, simulations showed that management based on molecular ancestry and IBD segments resulted in higher maintained genetic diversity and fitness as opposed to management based on a pedigree [21]. In the IBD segments management scenario the length of the segments was crucial for this result: in longer IBD segments there is a higher chance of homozygous deleterious alleles as these regions have a common ancestor [21]. By using genomic information in a breeding program, negative effects from inbreeding and deleterious load can be more actively avoided.

The results of the genomic analyses as presented in this study show that they can be of direct use for conservation management either* in situ* or* ex situ*, even with the small sample sizes generally available in conservation settings.* In situ *the identification of a substructure can lead to reassessing priorities for conservation. Identifying hybrids (as hybridization is a big threat to wildlife worldwide [37]) can give better insights into the effect of the threat and can help select individuals for (captive) breeding programs. In addition, an analysis of historic population size can explain levels of nucleotide diversity present in the population and put the current numbers in a historic perspective.* Ex situ,* the selection of founder individuals [38] and identification of relatedness between founder individuals can lead to more informed reproductive planning, resulting in higher levels of genetic diversity maintained. This can even be increased by assessing individual inbreeding levels or identifying carriers of deleterious alleles and incorporating this information in breeding recommendations [39]. To do this for all individuals in the current (captive) breeding programs is, for the time being, very time-consuming and costly. However, knowing this information from the founders, as well as being able to monitor and model the behaviour of the genetic material through the breeding program, is feasible. For example, if genomic information is known for the founders, genetic markers such as a subset of SNPS or microsatellites uniquely identifying the founders should suffice to adequately manage the next generations for inbreeding and deleterious variation. This combination of techniques is cost-efficient but does require information from the founders, which is not always available.

Another way of using genomic information without sequencing all individuals is to model the population and simulate what will happen with the genetic material under certain management strategies. There are already software programs available that can do just this. Examples are the PMx 2000 software [19], which monitors a population and can project future demographics under certain management strategies, and Vortex software [40] that uses demographic and stochastic factors to calculate an extinction risk of a population (a Population Viability Analysis, PVA). Some genetic information, such as inbreeding (modelled as a default value for deleterious load and the effect on juvenile mortality), can already be incorporated in these tools and thus be modelled over time. This information however is not species-specific and far from the detailed level of information that is available with genomic analyses [41]. New insights gained by genomic studies can provide more detailed input for these or similar software programs and can be used in concordance with other data sources [42, 43]. Incorporation of this information in management tools has been identified as a “conservation priority” [41] and can lead to less uncertainty and more successful breeding programs.

An example of a measure that could be of interest for these modelling efforts is the number and length of ROHs. It has already been found that the selection of individuals for participating in breeding programs based on ROHs gave the best results in maintaining diversity without losing much fitness, as compared to optimal contributions and including inbred matings (purging of deleterious variation) [44].

Although genomic data can already provide extremely useful information for the development of (conservation) management strategies, we need to understand more about the genomic measures and what they actually tell us about a population before they can be applied on a large scale. For example, about ROHs: how does the recombination landscape of the genome affect the distribution of ROHs over time? And how exactly does ROH-breakdown affect nucleotide diversity? Are these characteristics species-specific or is it possible to derive some general “rules-of-thumb”? With these uncertainties in mind, the incorporation of genomic analyses in conservation management may seem to be something of the distant future. Not least because the translation of academic knowledge to conservation practice is often slow [45]. However, the rate of development of sequencing technology has progressed very rapidly over the past decade and is expected to continue to do so, potentially enabling extremely cheap, whole genome variation information for all actively managed populations in the near future. Even today, it is already possible to use genomic analyses for individual cases of critically endangered species such as the Visayan warty pig in this example. Other examples include the California condor [46–48] and the North-American bison [49].

## 5. Conclusion

Genomic techniques represent a promising new toolset in the field of conservation biology. In this study genomic data analyses answered several questions regarding the captive population of the critically endangered Visayan warty pig. We found evidence for a recent split between the two island populations. However, with the current level of inbreeding, the viability of the total captive population and the probability of reintroduction might increase by merging the two captive populations. With the current rate of development, and associated lower costs, it is expected that genomic techniques will be feasible for broad application in conservation biology in the near future.

## Supplementary Material

Supplementary Material for Nuijten et al. 2016 including a description of all species (other than Sus cebifrons) used in this study (Table S1), an overview of the cross-validation errors for the Admixture analysis (Table S2), a presentation of shared and island specific variants found in Sus cebifrons individuals (Table S3) and the number of variants in homozygous or heterozygous state, both for synonymous, non-synonymous and non-synonymous, not-tolerated variants (Table S4). Figures include the results of a PSMC analysis for the other species included in this study for comparison (Figure S1), an overview of the coverage of regions of homozygosity both for Sus cebifrons (Figure S2) and for individuals of the other species (Figure S3) and the distribution of nucleotide diversity in Sus cebifrons individuals (Figure S4).

## Figures and Tables

**Table 1:** Sample codes of resequencing data available for seven *Sus cebifrons* individuals, two from Rotterdam zoo and five from San Diego zoo.

Rotterdam (Negros)	San Diego (Panay)
SCEB02M01	SCEB01F01
SCEB02M02	SCEBKb14130
	SCEBKb16637
	SCEBKb16508
	SCEBKb17528

**Table 2:** Overview of number of ROHs, average length of ROHs, and nucleotide diversity outside ROHs for all *Sus cebifrons* individuals and other *Sus* individuals. Description of other *Sus* species is in Supplementary Material, Table S1. Asian wild boars 1 and 2 originate from north China and south China, respectively. European wild boars 1 and 2 represent populations from Italy and France, respectively.

Sample code or species name	Sex	Inbreeding coefficient (*F*)	# ROHs (>20 bins)	Average length of ROHs (kb)	Nucleotide diversity outside ROHs (*π*)
02M01	M	0	34	761.43	12.5
02M02	M	0	75	1764.21	12.1
01F01	F	0.1875	110	2245.32	11.9
Kb16508	M	0.0625	132	1420.68	12.4
Kb16637	F	0	144	1860.76	12.4
Kb17528	F	0.25	193	2867.06	12.0
Kb14130	F	0	130	2563.13	12.2
*Sus verrucosus*	U	NA	275	3829.13	6.3
*Sus barbatus*	U	NA	11	1551.82	26.4
*Sus celebensis*	U	NA	36	2719.72	23.2
Asian domestic	M	NA	271	2796.31	30.9
Asian wild boar 1	U	NA	155	1966.39	28.9
Asian wild boar 2	U	NA	44	2120.00	33.9
Asian wild boar (Japan)	U	NA	1172	1573.41	17.5
European domestic	F	NA	493	1859.01	28.4
European wild boar 1	M	NA	592	1912.82	16.2
European wild boar 2	U	NA	708	1401.60	18.4

**Table 3:** Assessment of shared and island-specific variation. The vast majority of the SNPs, both coding and noncoding, are shared between islands, and only a very small portion may be specific. Note that this pertains a total of 4679012 SNPs, excluding fixed differences between *S. scrofa* and *S. cebifrons*, for which the minor allele count is at least two out of seven individuals, or 14 haplotypes.

	Shared	Panay (*N* = 5)	Negros (*N* = 2)
All (4679012 SNPs total)	3969361	457741	251910
Synonymous	19565	2150	1190
Nonsynonymous tolerated	10081	918	533
Nonsynonymous not-tolerated	3370	343	171
