# Supplementary material for: The Use of Genomics in Conservation Management of the Endangered Visayan Warty Pig (Sus cebifrons)
Source: Int J Genomics. 2016 Mar 16;2016:5613862. doi: 10.1155/2016/5613862 (PMC4812450; doi:10.1155/2016/5613862)
Supplement: Supplementary file 1 — Supplementary Material for Nuijten et al. 2016 including a description of all species (other than Sus cebifrons) used in this study (Table S1), an overview of the cross-validation errors for the Admixture analysis (Table S2), a presentation of shared and island specific variants found in Sus cebifrons individuals (Table S3) and the number of variants in homozygous or heterozygous state, both for synonymous, non-synonymous and non-synonymous, not-tolerated variants (Table S4). Figures include the results of a PSMC analysis for the other species included in this study for comparison (Figure S1), an overview of the coverage of regions of homozygosity both for Sus cebifrons (Figure S2) and for individuals of the other species (Figure S3) and the distribution of nucleotide diversity in Sus cebifrons individuals (Figure S4). [file 5613862.f1.docx]

**Supplementary Information**

| **Table S1**. Other individuals used to compare our results with. The different species/populations all have their own demographic history and thus their own interpretation of results and the comparison. | |
| --- | --- |
| **Species** | **Description** |
| *Sus verrucosus* | Javan warty pig, lives in Indonesia (Java), this sample is from inbred zoo animal, originated from very small source population. |
| *Sus barbatus* | Bearded pig, lives in Indonesia and Malaysia, probably large outbred population present. |
| *Sus celebensis* | Sulawesi warty pig, lives in Indonesia (Sulawesi), probably large outbred population present. |
| *Sus scrofa –* Asian domestic | Domestic breed (Meishan) originated from Asian wild boar, limited population size. |
| *Sus scrofa –* Asian wild | Asian wild boar, large outbred population for long time, these samples are from North and South China. Sample from Japan experienced very severe bottleneck but recovered. |
| *Sus scrofa –* European domestic | Domestic breed (Large White) originated from European wild boar but admixed with Chinese domestic breeds. |
| *Sus scrofa –* European wild | European wild boar, originated from Asian wild boars, experienced severe bottleneck, currently fragmented population. |

| **Table S2.** Cross-validation errors for all possible K-values in the Admixture analyses. K = 6 had the lowest cross-validation error. We used K = 2 because of prior knowledge on the origin of the samples. | |
| --- | --- |
| **K-value** | **Cross-validation error** |
| 1 | 1.35 |
| 2 | 1.44 |
| 3 | 1.93 |
| 4 | 1.36 |
| 5 | 0.60 |
| 6 | 0.41 |


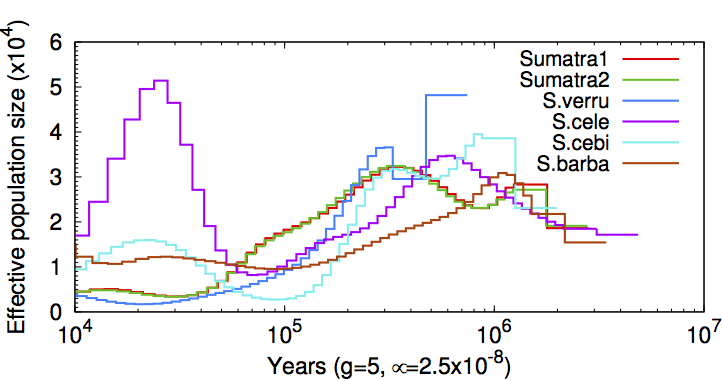


**Figure S1**. Estimated effective population size of other *Sus* species based individual genomes, generation time (i.e. 5 years) and mutation rate (2.5*10^-8^) from 10.000 (left) to 1.000.000 (right) years ago (Frantz et al. 2013). All species experienced a bottleneck to some extent around 100.000 years ago. Sumatra1 and Sumatra 2 are wild boar populations (*Sus scrofa*) from Sumatra.

**Figure S2**. Total coverage as proportion of the genome of all ROHs (left of dotted line) and of ROHs per length category (right of dotted line).

**Figure S3**. Total coverage as proportion of the genome of all ROHs (left of dotted line) and of ROHs per length category (right of dotted line). Counter to what logic predicts, not all individuals have the largest proportion of the genome covered by the longest ROHs. Many short ROHs are a sign of severe inbreeding in the past. Species description in Table S1. WB29U14 = Wild Boar South China, WB30U08 = Wild Boar North China, WB20U02 = Wild Boar Japan.

**Figure S4.** Proportion of bins (10kb) per category of SNPs per bin. On average 12 SNPs per bin were found in *Sus cebifrons*, 23 SNPs per bin were found on average in the other individuals (Table 2).

| **Table S3.** Assessment of shared and island-specific variation. SNPs in coding regions only. Fixed differences between *Sus cebifrons* and *Sus scrofa* were excluded. The vast majority of the SNPs is shared between islands, and only a very small portion may be specific. | | | | | |
| --- | --- | --- | --- | --- | --- |
|  | **Shared** | **Panay**  **(N = 5)** | **Negros**  **(N = 2)** | **Total** | **Fraction island specific variation** |
| **All** | 3969361 | 457741 | 251910 | 4679012 | 0.152 |
| **Synonymous** | 19565 | 2150 | 1190 | 22905 | 0.146 |
| **Non-synonymous tolerated** | 10081 | 918 | 533 | 11532 | 0.126 |
| **Non-synonymous not-tolerated** | 3370 | 343 | 171 | 3884 | 0.132 |

| **Tabls S4.** Number of variants in homozygous (Hom) or heterozygous (Het) state, per category (All, synonymous, non-synonymous tolerated and non-synonymous not-tolerated). The ratio represents the Het/Hom ratio. This ratio is increased in not-tolerated variants, suggesting some degree of purging has taken place. | | | | | | | | | | | | |
| --- | --- | --- | --- | --- | --- | --- | --- | --- | --- | --- | --- | --- |
|  | **All_variants** | | | **Synonymous_variants** | | | **Non-synonymous Tolerated_variants** | | | **Non-synonymous Not-tolerated_variants** | | |
|  | **Hom** | **Het** | **Ratio** | **Hom** | **Het** | **Ratio** | **Hom** | **Het** | **Ratio** | **Hom** | **Het** | **Ratio** |
| **02M01** | 3067603 | 1611409 | 0.525 | 14157 | 8748 | 0.618 | 6808 | 4724 | 0.694 | 2258 | 1626 | 0.720 |
| **02M02** | 3141793 | 1537219 | 0.489 | 14492 | 8413 | 0.581 | 6926 | 4606 | 0.665 | 2277 | 1607 | 0.706 |
| **01F01** | 2847575 | 1831437 | 0.643 | 13350 | 9555 | 0.716 | 6425 | 5107 | 0.795 | 2076 | 1808 | 0.871 |
| **Kb14130** | 2836079 | 1842933 | 0.650 | 13403 | 9502 | 0.709 | 6357 | 5175 | 0.814 | 2057 | 1827 | 0.888 |
| **Kb16508** | 2633978 | 2045034 | 0.776 | 12265 | 10640 | 0.868 | 5849 | 5683 | 0.972 | 1911 | 1973 | 1.032 |
| **Kb16637** | 2635637 | 2043375 | 0.775 | 12440 | 10465 | 0.841 | 5893 | 5639 | 0.957 | 1923 | 1961 | 1.020 |
| **Kb17528** | 2829669 | 1849343 | 0.654 | 13239 | 9666 | 0.730 | 6227 | 5305 | 0.852 | 1986 | 1898 | 0.956 |
